# Comparison of dissolved and particulate arsenic distributions in shallow aquifers of Chakdaha, India, and Araihazar, Bangladesh

**DOI:** 10.1186/1467-4866-9-1

**Published:** 2008-01-11

**Authors:** Jerome Métral, Laurent Charlet, Sara Bureau, Sukumar Basu Mallik, Sudipta Chakraborty, Kazi M Ahmed, MW Rahman, Zhongqi Cheng, Alexander van Geen

**Affiliations:** 1 Environmental Geochemistry Group, LGIT-OSUG, University of Grenoble, BP 53, F-38041 Grenoble, Cedex 9, France; 2 Department of Geological Sciences, Jadavpur University, Calcutta, India; 3 Department of Chemistry, Kalyani University, West Bengal, India; 4 Department of Geology, University of Dhaka, Dhaka 1000, Bangladesh; 5 Lamont-doherty EarthObservatory of Columbia University, Palisades, NY 10964, USA

## Abstract

**Background:**

The origin of the spatial variability of dissolved As concentrations in shallow aquifers of the Bengal Basin remains poorly understood. To address this, we compare here transects of simultaneously-collected groundwater and aquifer solids perpendicular to the banks of the Hooghly River in Chakdaha, India, and the Old Brahmaputra River in Araihazar, Bangladesh.

**Results:**

Variations in surface geomorphology mapped by electromagnetic conductivity indicate that permeable sandy soils are associated with underlying aquifers that are moderately reducing to a depth of 10–30 m, as indicated by acid-leachable Fe(II)/Fe ratios <0.6 in the solid phase and concentrations of dissolved sulfate >5 mg L^-1^. More reducing aquifers are typically capped with finer-grained soils. The patterns suggest that vertical recharge through permeable soils is associated with a flux of oxidants on the banks of the Hooghly River and, further inland, in both Chakdaha and Araihazar. Moderately reducing conditions maintained by local recharge are generally associated with low As concentrations in Araihazar, but not systematically so in Chakdaha. Unlike Araihazar, there is also little correspondence in Chakdaha between dissolved As concentrations in groundwater and the P-extractable As content of aquifer particles, averaging 191 ± 122 ug As/L, 1.1 ± 1.5 mg As kg^-1 ^(n = 43) and 108 ± 31 ug As/L, 3.1 ± 6.5 mg As kg^-1 ^(n = 60), respectively. We tentatively attribute these differences to a combination of younger floodplain sediments, and therefore possibly more than one mechanism of As release, as well as less reducing conditions in Chakdaha compared to Araihazar.

**Conclusion:**

Systematic dating of groundwater and sediment, combined with detailed mapping of the composition of aquifer solids and groundwater, will be needed to identify the various mechanisms underlying the complex distribution of As in aquifers of the Bengal Basin.

## Background

**Figure 1:**
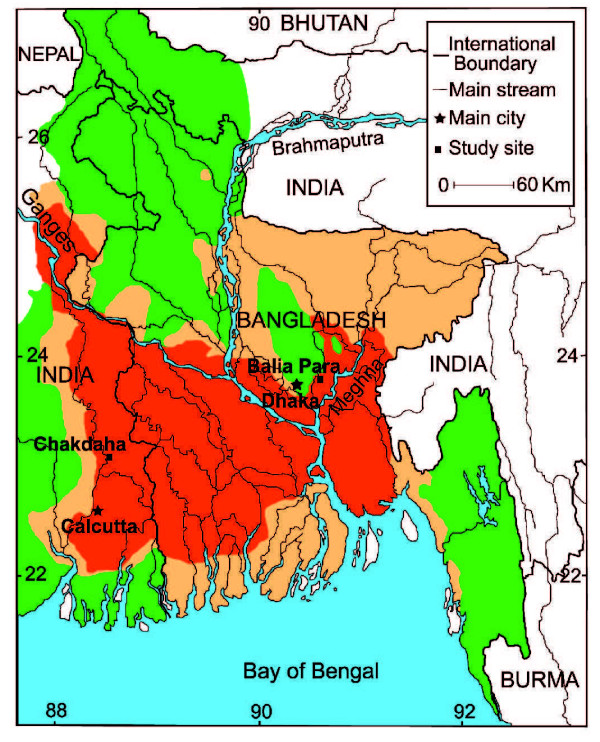
Map of the entire Bengal Basin showing the general distribution of As in tubewells on the basis of data collected by Dr. Chakraborti's laboratory at the School of Environmental Studies, Jadavpur University, Kolkata, India. Most tubewells contain >50 μg L^-1 ^arsenic in red areas and <50 μg L^-1 ^in green areas. Orange areas indicate a mixed distribution high- and low-As well. Also shown are the location of the two study sites and the two largest cities in the region. Adapted from [9].

Following the attribution of skin lesions affecting villagers of West Bengal (India) to arsenicosis in the mid 1980's, studies of the groundwater As problem have been conducted throughout the Bengal Basin across a range of spatial scales. Most of the initial efforts have focused on mapping the extent of the problem by testing the As content of a subset of existing tube wells over large areas at the ~10–100 km scale [1,2] (Fig. 1). This pioneering work demonstrated that some areas were clearly more affected than others at the regional scale, while at the same time indicating significant variability at smaller spatial scales. In parallel, the vertical dimension of the problem began to be addressed by drilling and the purposeful installation of nests of piezometers [2]. Variations in the composition of groundwater and aquifer solids for constituents other than As were documented in an attempt to identify the processes responsible for As mobilization [2-5]. Incubations were conducted using aquifer material recovered during drilling under conditions that, however, were typically different from the original conditions in the subsurface [6,7]. Despite these multiple approaches and nearly a decade of field and laboratory work conducted by various groups of scientists, a consensus has yet to emerge with respect to the most basic factors that lead to elevated As concentrations in groundwater of the Bengal Basin. A key issue such as the source of organic matter that drives Bengal delta aquifers enriched in As towards reduction is still disputed [5,8,9]. Moreover, recent work has shed doubt on microbial reduction of Fe oxhydroxides as the only cause of As mobilization and on the depth at which As mobilization actually occurs [10,11].

In this paper, we argue that variations in aquifer properties on lateral scales of ~100 m must be considered to understand the process of As mobilization in relatively shallow (<20 m) aquifers of Holocene age. These are the aquifers tapped by a majority of existing private wells; groundwater in this depth range also encompasses the widest range of As concentrations [2]. The primary objective of the present study was to determine if recent findings from Araihazar, Bangladesh, indicating that interactions between geology and hydrology at the 10–100 m scale influence As concentrations in shallow aquifers [12-14], were applicable to other regions of the Bengal Basin. We wanted to know, in particular, if the distribution of As in groundwater of Chakdaha, India, could be re-interpreted in terms of variations in local hydrogeology instead of a plume emanating from a "hot spot" [15-18]. An inexpensive device that relies on the manual drilling method to install wells in both West Bengal and Bangladesh [19] was used to collect detailed transects of groundwater and aquifer solid properties. We also present here surface geophysical data to characterize the nature of surface soils in the areas surrounding the two transects. The next two sections describe the main features of the two study areas and the methods used to collect and analyze aquifer material. The geophysical and geochemical observations are first described with relatively little interpretation. In the subsequent discussion, we examine some of the relationships between various geochemical parameters in the light of patterns of groundwater recharge suggested by variations in the nature of surface soils. We conclude by exploring the reasons why a relationship between groundwater As concentrations and the P-extractable As content of the sediment documented in Bangladesh does not seem to hold for floodplain aquifers along the Hooghly River.

### Geological setting

**Figure 2:**
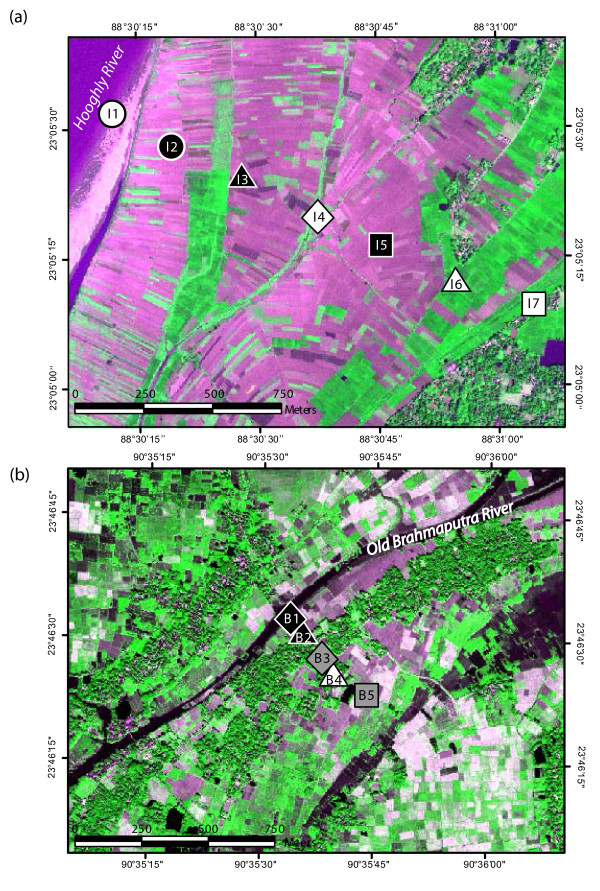
IKONOS satellite images of study areas in (a) India and (b) Bangladesh acquired in April 2000 and November 2000, respectively. The images were processed to emphasize in purple the water contained in streams, ponds, and irrigated rice paddies. Mottled areas indicate trees, which typically correspond to where dwellings are located. Also shown are symbols indicating the positions of needle-sampler profiles obtained in the two study areas. Unlike subsequent figures, the scale is the same for both images.

**Figure 3:**
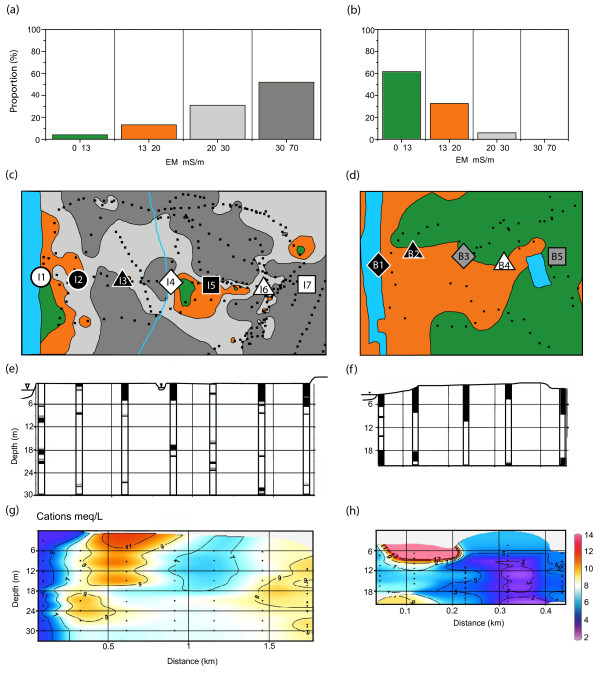
Histogram of EM conductivities around (a) Chakdaha, India and (b) Balia Para, Bangladesh. The location of the measurements is shown by black symbols on a contour map of EM conductivity, color-coded in the same ranges as in the histogram (c-d). Also shown are the locations of needle-sampler profiles using the same symbols of Fig. 2. (e-f) Lithological cross-sections for the two sites showing the distribution of clay/silt (black) and sand (white). (g-h) Contoured section of major cation concentrations for groundwater collected with the needle-sampler. Sections drawn with Ocean Data View [47].

Previous studies have been conducted in both areas selected for this comparison. Chakdaha block, 65 km to the north of Kolkata, is located in the Ganges River delta floodplain and bordered on the west side by the Hooghly River, the largest tributary of the Ganges in West Bengal, India (Fig. 1). Groundwater pumped from 235 tube wells distributed over a 19 km^2 ^area and ranging in depth from 5 to 200 m has been analyzed for As and major ions in the laboratory [15-17]. Concentrations of As in roughly half the sampled wells exceed the Indian standard for drinking water of 50 μg L^-1^, with the highest levels (up to 400 μg L^-1 ^As) restricted to the 10–40 m depth range. The 1800-m transect of groundwater and sediment properties presented here extends from the bank of the Hooghly River to the rim of Chakdaha village, where a hot-spot in groundwater As at a depth of ~30 m has been documented [15-17,19] (Fig. 2a). A small stream parallel to the Hooghly flows south roughly mid-way across the floodplain deposits that separate the village from the river bank. The relative elevation of the drill sites was not measured in India, but the appearance of the topography is very flat. The only exception is Chakdaha village at the eastern end of the transect, which is elevated by about 2–3 m relative to the adjacent fields (Fig. 3e). The entire area of cultivated fields crossed by the transect is flooded during the monsoon.

The setting of the study in Araihazar upazila, Bangladesh, 30 km northeast of Dhaka, is comparable in some ways. A blanket survey of groundwater As has shown that in this region as well more than half the ~6000 wells distributed over a 25 km^2 ^area do not meet the Bangladesh standard for drinking water of 50 μg L^-1^As, with high levels (up to 800 μg L^-1^) concentrated in the 10–30 m depth interval [20]. The Old Brahmaputra River passing through the area is considerably smaller than the Hooghly River, however, and stops flowing during the dry season. The brown color of its water during the dry season suggests elevated dissolved organic matter concentrations. Supply of sediment to the area is very limited today, although there is evidence of a much larger stream, possibly a tributary of the Brahmaputra River, flowing through the area until a few hundred years ago [12]. The 450-m transect of groundwater and sediment properties obtained in Araihazar extends from the bank of the Old Brahmaputra River, through Balia Para village, and ends in a low-lying expanse of fields located in the partially filled channel of a former river (Fig. 2b). Concentrations of As in wells <25 m deep are particularly elevated (253 ± 132 μg L^-1^, n = 75) in Balia Para. There is a fairly large (30 m × 70 m) rectangular pond located just east of the village. The relative elevation of the drill sites in Bangladesh was measured within ± 1 cm using a transparent water tube filled with water extended from one drill site to the next. Here too, the village is elevated ~2 m relative to the adjacent cultivated fields, but is located in the middle of the transect rather than at the end of the transect as in Chakdaha (Fig. 3f). The two drill sites in low lying-areas at both ends of the transect crossing Balia Para are always flooded during the monsoon, whereas the sites in between are typically elevated enough to prevent this from happening.

A number of factors control a complex pattern of seasonal groundwater recharge, discharge, and flow reversals in shallow aquifers of the Bengal Basin. During the dry season, which extends from October through May, hydraulic heads generally favor discharge from the shallow aquifers into local streams and rivers [2,5,14,21]. At the onset of the monsoon in June, however, water levels rapidly rise by several meters and aquifers are recharged through a combination of vertical infiltration of precipitation and flood water, as well as lateral infiltration through river banks. ^3^H-^3^He dating of groundwater conducted in Araihazar has demonstrated that the rate of vertical recharge varies widely from 0.1–1 m/yr between neighboring villages as a function of the permeability of surface soils in surrounding fields [13,14]. Groundwater flow in shallow aquifers of the Bengal Basin may also be locally influenced during the dry season by recharge from persistent elevated ponds as well as uptake by the roots of trees that are typically concentrated in villages [21].

Despite the large number of hand-pumped tubewells in both study areas, groundwater withdrawals are likely to be dominated by mechanized pumping [5,21]. In Chakdaha, a cone of depression in water levels ~1 km east of the transect during the dry season has been attributed to pumping for a public water supply system in the center of Chakdaha [16]. Variations in groundwater levels in a network of wells distributed across the village over the seasonal cycle indicate net flow from the Hooghly River towards the east, within a regional hydraulic gradient that slopes gently towards the southeast. In Bangladesh, mechanized irrigation pumping is particularly intense in the fields east of Balia Para [13] and probably controls groundwater flow during the dry season. Water levels have not been monitored in Balia Para, but time series of hydraulic head at other locations within Araihazar indicate annually-averaged discharge towards the Old Brahmaputra River or other local depressions in topography [14].

## Methods

### Surface electromagnetic (EM) conductivity survey

Geophysical surveys of the 2 km^2 ^area surrounding the transect in India and the 0.2 km^2 ^area surrounding the shorter transect in Bangladesh were conducted using a Geonics^® ^EM31 instrument deployed horizontally [22-26] (see additional file 1). The instrument measures the interactions between the ground and an electromagnetic field generated by a transmitter coil. In theory, 50% of the signal is generated in the upper 90 cm of the soil; conductivity of layers below 180 cm depth still accounts for 27% of the signal [22,26]. Aziz et al. [13] compared EM conductivity measurements over a broad area of Araihazar with the properties of soil collected with a hand-auger and concluded that the EM conductivity signal is determined by the clay content of the soil and the concentration of major ions in soil water. Conductivities in the 20 to 40 mS m^-1 ^range were typically observed over clayey soils with an elevated major ion content and conductivities <10 mS m^-1 ^above sandy soils with a low major ion content.

### Water and sediment sampling

Unconsolidated deposits were drilled in India and Bangladesh using the manual "hand-flapper" method with 3-m sections of PVC or galvanized iron (GI) pipe [27,28]. A total of 7 boreholes ~250 m apart were drilled to a depth of 30 m along the Indian transect. In Bangladesh, the 5 boreholes are only ~125 m apart and extend to a depth of ~20 m. During drilling, variations in basic lithology (sand, silt, or clay) were recorded on the basis of the borehole washings. At depth intervals of 2–4 m, the drilling was interrupted to deploy the needle-sampler for collecting a total of 104 slurry samples (~100 mL) of groundwater and sediment from a depth ~0.3 m below the bottom of the drill hole (see additional file 1). An additional set of 21 needle-samples was collected only 0.3–1 m deeper than the previously sampled interval to test reproducibility. Immediately after collection, the headspace of the needle-sampler was purged with N_2_. About 5–10 mL of groundwater contained in the needle-sampler was then filtered under a gentle N_2 _pressure through a 0.45 μm syringe filter and into acid-leached polyethylene scintillation vials (PolySeal cap). Another aliquot of groundwater was filtered into scintillation vials that had been rinsed with MQ water only. Sediment contained in the N_2 _purged needle-sampler was stored in the dark until further processing on the evening of collection. With the exception of 2 profiles previously obtained in Bangladesh in January 2003 [29], all samples were collected between March and May 2005. The conductivity of groundwater samples collected in 2005 was measured with a calibrated TetraCon WTW probe on the day of collection. Water from the Hooghly River and the Old Brahmaputra River collected in 2005 was also filtered through a 0.45 μm syringe filter and stored in acid-leached and MQ-rinsed scintillation vials. Surface or well water used for drilling and water overflowing from the drill hole was also sampled and filtered to detect potential sampling artifacts. Finally, groundwater samples were collected from an additional 39 tube wells in India after pumping at a rate of 20–35 L min^-1 ^for at least 5 min.

### Solid phase extractions

Sediment collected with the needle-sampler at 2–4 m intervals was subjected to two types of treatment. The first was a 10% hot HCl extraction that is likely to release by dissolution As bound to amorphous and labile crystalline Fe oxyhydroxides phases as well as, potentially, As coprecipitated with acid-volatile sulfides [30,32]. On the evening of sampling, the proportion of Fe(II) and Fe(III) contained in the HCl leach was measured spectrophotometrically with ferrozine [28,30]. The second extraction takes place over 24 hours and uses a 1 M Na_2_HPO_4 _(pH = 5) solution, purged with N_2_, to dislodge by anion exchange the relatively mobile fraction of As bound to Fe oxhydroxides and, potentially, As bound to adsorbed humic acids [33-35].

### Chemical analyses

At least 1–2 days before analysis, groundwater and river samples stored in acid-leached scintillation vials were acidified to 1% HCl (Optima, Fisher Scientific) to ensure re-dissolution of Fe oxhydroxides that might have precipitated. This method of delayed acidification was validated by comparing the composition of tube well samples stored for 11 months with replicates that had been acidified in the field [36]. All water samples were diluted 1:10 in 1% HNO_3 _(Optima, Fisher Scientific) and analyzed for As, Ca, Fe, K, Mg, Mn, Na, and S by high-resolution inductively coupled plasma mass spectrometry (HR ICP-MS) at Lamont-Doherty Earth Observatory of Columbia University. The method has a detection limit of ~1 μg L^-1 ^for As and a precision of ~5% for all constituents [37]. Sediment leachates were also analyzed by HR ICP-MS following 1:100 dilution for As and Fe. Water samples stored in scintillation vials that were not acid-leached were analyzed for Cl^-^, Br^-^, F^-^, NO_3_^-^, and SO_4_^2- ^by ion chromatography (Dionex DX500 and a Dionex separation column). The method has a detection limit ~0.1 mg L^-1 ^and a precision of ~ 10% for all anions. With the exception of one outlier, S determinations by HR ICP-MS and SO_4_^2- ^by ion chromatography were essentially identical [see Additional file 2, Fig S1]. The alkalinity of groundwater collected with the needle-sampler was measured by titration at the end of each of sampling day.

## Results

### Lithology

Drilling to a depth of ~30 m at sites I1- I7 showed that the lithology of the floodplain of the Hooghly River near Chakdaha is quite variable. Surface clay layers ranging from 1 to 6 m in thickness were recorded at 5 out of 7 sites (Fig. 3e). At sites I1 and I5, instead, coarse to fine sand deposits similar to the deeper aquifer material extended all the way to the surface. The aquifer material was grey in color at all sites and occasionally interrupted by 0.3–1 m lenses of fine silt or clay at all drill sites.

In Bangladesh, the drillers had to work through a 4–10 m thick surface clay layer at all five sites before reaching grey aquifer sands (Fig. 3f; note that B2 and B4 have been previously described as NS7 and NS8 [29]). Drilling was stopped at all sites in Balia Para when a second thick clay layer was encountered at 18–20 m depth. Clay lenses were not encountered within the sandy aquifers, with the exception of two ~1-m thick intervals at B1. The aquifer material recovered from Bangladesh was also always grey in color.

### Spatial patterns of EM conductivity

Electromagnetic conductivity was measured in India at ~300 locations distributed over a ~2 km^2 ^area and ranged from 8 to 63 mS m^-1 ^(average = 31 ± 11 mS m^-1^). Over half the readings in India exceeded 30 mS m^-1 ^(Fig. 3a). The spatial density of sampling was sufficient to reveal a ~200 m swath of conductivities <20 mS m^-1 ^along the banks of the Hooghly River and another pocket of low EM conductivity further inland near I4 and I5 (Fig. 3c). Drill holes I1 and I5, where sandy deposits were observed to extend all the way to the surface, are both located within areas of relatively low EM-conductivity.

The density of dwellings constructed of corrugated iron plates and overhead power lines limited the collection of EM conductivity data to ~100 readings over the ~0.2 km^2 ^study area in Bangladesh. The EM measurements span a narrower range of 8–27 mS m^-1 ^(Fig. 3b). Even though low EM conductivities in Araihazar are associated with sandy surface soils and no such deposits were encountered during drilling across Balia Para, the average EM conductivity (12 ± 4 mS m^-1^) in Bangladesh is more than a factor of two lower than in India, with over half the readings <13 mS m^-1^. Another difference between the two study areas is that EM conductivities do not decline on the banks of Old Brahmaputra River, unlike EM conductivities close to the Hooghly River (Fig. 3d).

### Reproducibility of needle-sampling

Profiles of groundwater properties have been collected close to several nest of monitoring wells to demonstrate the reliability of the needle-sampler [19]. True replication as an additional test is not possible because the same depth interval cannot be sampled twice, however. The risk is perturbation of the groundwater, not the sediment, because deployment of the device within a PVC pipe essentially eliminates contamination of the bottom of the hole with sediment material falling from a shallower depth [19]. The drilling procedure requires the hole to be entirely filled with water and therefore sets up a hydraulic head in the hole at the end of dry season, when the samples were collected, that is ~5 m higher than the water table of the underlying aquifers. Timing is therefore of the essence because hole water will eventually migrate to the depth sampled by the needle. Fortunately, such contamination can typically be detected because hole water is laden with clay particles that remain in suspension. We have occasionally observed clay particles in water collected by the needle-sampler and have attributed their presence to migration of groundwater below the bottom of the hole or to an early trigger of the sampler. This can be confirmed independently by comparing the composition of hole water for relatively unreactive constituents such as the major cations. Of the set of 104 samples collected at 2–4 m interval, only one outlier from I1 at 19 m depth was eliminated from further consideration because its groundwater composition was similar to that of hole water.

The 21 pairs of samples collected within 0.3–1 m of each other in India and Bangladesh provide a measure of replication, even if spatial variability of the composition of groundwater over very short vertical distances cannot be disentangled. In the case of Ca, Mg and Si concentrations spanning the 10–30 mg L^-1 ^range, the correspondence between such "replicates" is remarkable, and not merely because the profiles at a particular location are invariant [See Additional file 2, Fig 2a and 2b]. The agreement between pairs of samples is somewhat weaker for Na and K, suggesting that water-rock interactions (e.g. micas) result in greater spatial variability in groundwater composition for the monovalent cations. The agreement for paired samples is encouraging also in terms of sulfate and Mn concentrations [See Additional file 2, Fig 2c and 2d]considering that redox reactions could produce a very heterogeneous distribution of groundwater properties in the subsurface. Most importantly, there is a good correspondence between As concentrations measured for the set of paired samples [See Additional file 2, Fig 2f]. Deviations from a one-to-one relationship are typically consistent with an increase of As concentrations with depth, which is indeed often observed in shallow aquifers [2,5,19,28,34]. In contrast to concentrations of the major cations, sulfate, Mn, and As, there is little correspondence between concentrations of Fe for pairs of samples collected 0.3–1 m apart [See Additional file 2, Fig 2e]. We believe this is a reflection of particularly pronounced spatial variability, although a sampling artifact cannot be ruled out with absolute certainty. The syringe filters typically turn light brown and eventually clog, but we attribute this to trapping of fine-grained particles rather than Fe precipitation. For constituents other than Fe, there is therefore little doubt that the spatial variability documented with the needle-sampler is real and not merely a sampling artifact.

### Major cations in river and groundwater

River and groundwater of the Bengal Basin is typically circum-neutral (pH = 7.0 ± 0.5) and this also the case for the two study areas [2,16,34]. The combined concentrations (in equivalents) of the major cations Ca, K, Mg, and Na therefore provide a useful proxy of conductivity and the concentration of total dissolved solids [See Additional file 2, Fig. S3]. Because conductivity was not measured in groundwater at sites B2 and B4 sampled in January 2003 in Bangladesh, we discuss hereon only the combined concentration of major cations. The concentration of major cations in both the Hooghly and Old Brahmaputra rivers (3 and 4 meq L^-1^, respectively) is lower than for any of the groundwater that was analyzed. In India, the combined concentration of major cations in groundwater is relatively low (<8 meq L^-1^) at the site closest to the Hooghly River (I1) and below the patch of low EM conductivity further inland (I4 and I5; Fig. 3c,g). At two of these sites (I1 and I5), a thick surficial clay layer was not encountered during drilling. In Bangladesh, there is also a broad spatial association between surface EM conductivity and major cation concentrations in shallow groundwater. Major cation concentrations in groundwater are relatively low (<6 meq L^-1^) inland at B4-B5 where lower EM conductivities were also recorded (Fig. 3d,h). Major cation concentrations are low at B1 on the banks of the Old Brahmaputra River, but in this case not accompanied by a decrease in EM conductivity.

### Arsenic in river and groundwater

Of the total of 62 intervals sampled 1.5–3 m apart vertically in India and 42 similarly-spaced intervals in Bangladesh, essentially the same proportion of groundwater samples contains 50–200 μg L^-1 ^As (44 and 43%, respectively). The two study sites clearly differ at the low and at the high end of As concentrations, however. The proportion of samples collected along the two transects containing <50 μg L^-1 ^and >200 μg L^-1 ^is roughly balanced in India (27 and 29%, respectively), whereas in Bangladesh the number of samples containing <50 μg L^-1 ^As is only one quarter the number containing >200 μg L^-1 ^(12 and 45%, respectively).

**Figure 4:**
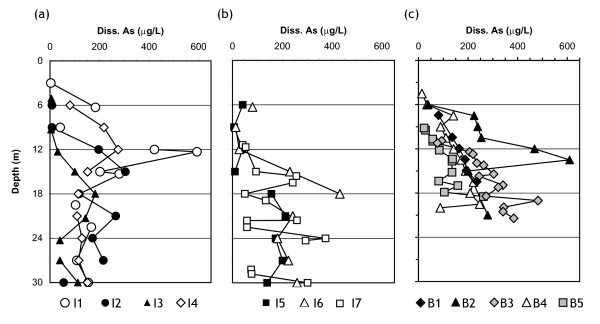
Vertical profiles of groundwater As concentration in (a-b) India, (c) Bangladesh. Symbols within the same profile are not connected if two samples were separated by an impermeable clay lens. Symbols identifying the profiles are the same as in Figure 2.

**Figure 5:**
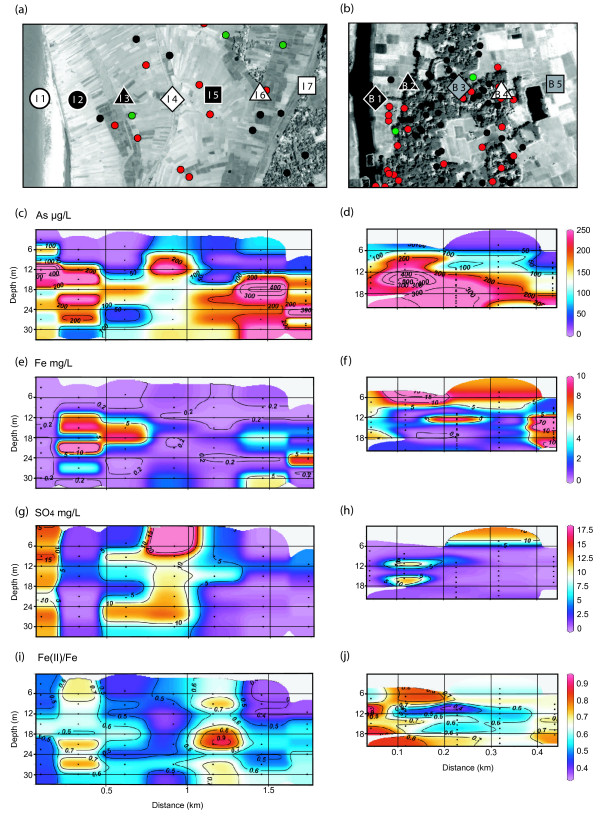
Distribution of dissolved As in groundwater (0–50 μg L^-1 ^in green; 50–200 μg L^-1 ^in red; and >200 μg L^-1 ^in black) from shallow tubewells shown on enlarged and rotated portions of the IKONOS images in Figure 2 for (a) Chakdaha, India and (b) Balia Para, Bangladesh. Also shown are contoured sections based on needle-sampler profiles for dissolved As (c-d), Fe (e-f), SO_4 _(g-h), and acid-leachable Fe(II)/Fe in the aquifer particles. Sections drawn with Ocean Data View [47].

Concentrations of As in filtered water from the Hooghly and the Old Brahmaputra rivers are low at 6 μg L^-1^. The highest As concentrations in India (~400 μg L^-1^) were encountered on the banks of the Hooghly River at I1 (12 m depth) and 1500 m inland at I6 (18 m), towards the location of the previously identified hot-spot [15-18] (Fig. 4a,b and Fig. 5c). Concentrations of As are also generally elevated in the nearby profiles I2 and I7 at either extremities of the transect. With the exception of the shallowest sample at I4, As levels in shallow groundwater are generally lower in the middle portion of the Hooghly River floodplain (I3-I5).

At all five sites in Bangladesh, As concentrations increase gradually with depth starting from a level of 10–90 μg L^-1 ^in the interval sampled closest to the surface (Fig. 4c, Fig. 5d). The highest As concentrations of 500–600 μg L^-1 ^were measured in Bangladesh at B2, 100 m inland of the Old Brahmaputra River. Within a depth interval, As concentrations generally decline with increasing distance east of B2. At B1, B3, and B5, the highest As concentration is reached in the last sample collected above the deep clay layer. The profile of As closest to the Old Brahmaputra (B1) is very similar to B3-B5 at the other end of the transect.

There is a striking difference in the depth distributions of As in India and Bangladesh. Whereas the shallowest samples from India also all contain <100 μg L^-1 ^As, none of the profiles from the floodplain of the Hooghly River show a steady increase in As concentrations with depth (Fig. 4a,b). Particularly at I1 and I7, As concentrations are highly variable from one sample to the next and throughout the entire depth range. In Bangladesh, the 200 μg L^-1 ^contour of As concentrations delineates what appears to be a contiguous volume of contaminated groundwater that extends from below a depth of ~10 m near the river (B1-B2) and is restricted to a depth below ~18 m inland at B5. There is no evidence for such continuity in the As data from India, but instead sharp shifts in concentrations beneath each clay layer or lens at B2 and B5, and to a lesser extent, at B4. The differences in heterogeneity could be due to the numerous clay lenses identified in India and their rare occurrence in Bangladesh. Possibly also because of the 3-fold longer distance between adjacent profiles, there is considerably less lateral continuity in the distribution of groundwater As concentrations in India (Fig. 5c).

### Redox-sensitive constituents of river and groundwater

The distribution of sulfate in India and Bangladesh is almost the mirror image of the distribution of major cations. Sulfate concentrations are elevated in both the Hooghly and Old Brahmaputra rivers at 17 and 14 mg L^-1^, respectively. In groundwater, sulfate concentrations exceed 10 mg L^-1 ^to a depth >20 m only near the Hooghly River at I1 and at I4, one of the two profiles located further inland within the patch of low EM conductivity (Fig. 5g). In Bangladesh as well, concentrations of sulfate >10 mg L^-1 ^are associated with low concentrations of major cations at several depths between 10–18 m at B2 and at very shallow depths at B4 (Fig. 5h). The overall proportion of samples with sulfate concentrations exceeding 2 mg L^-1 ^is considerably lower in Bangladesh than in India, however (5 out of 39 vs. 36 out of 60 samples analyzed by chromatography, respectively).

Filtered water from both the Hooghly and Old Brahmaputra rivers contains low but detectable levels of Fe (0.05 and 0.36 mg L^-1^, respectively). The highest concentrations of Fe measured in groundwater were comparable in India (16 mg L^-1^; 21 m depth at I2) and Bangladesh (18 μg L^-1^; 6 m depth at B2). The average concentration of Fe in all groundwater samples from India (1.7 ± 3.2 mg L^-1^) is about half the average for Bangladesh (3.9 ± 4.9 mg L^-1^), however. There is only a very general correspondence between the distribution of major cations and Fe concentrations in groundwater in India, and therefore an inverse relation to the distribution of sulfate (Fig. 5e). Major cation and Fe concentrations are low (<0.5 mg L^-1^) to a depth of 30 m near the Hooghly River (I1) and underneath the patch of low EM conductivity further inland (I4-I5). Conversely, Fe concentrations are occasionally >5 mg L^-1 ^at I2-I3 and I6-I7, although not necessarily at the depth corresponding to elevated major cation concentrations (Figs. 2, 4).

Unlike in India, there is pattern of generally decreasing dissolved Fe concentrations with depth across the transect in Bangladesh, with the exception of B5 (Fig. 5f). There is no systematic relationship between dissolved Fe and major cation concentrations, however. Major cation and Fe concentrations are both elevated in the upper portion of the profile at B2, but Fe concentrations are also elevated at depth in groundwater with <5 meq L^-1 ^in major cations at B5.

### Sediment properties

In both India and Bangladesh, leachable Fe(II)/Fe ratios in the sediment span the 0.3–0.9 range. Fe(II)/Fe ratios never drop below 0.2, the value characteristic of older deposits of orange-brown sands associated with As concentrations <10 μg L^-1 ^[28,34]. The proportion of samples with Fe(II)/Fe <0.5 is considerably larger in India (23 out of 62 samples) than in Bangladesh (2 out of 42), suggesting a generally less reducing environment in India.

Lower leachable Fe(II)/Fe ratios are concentrated in India near the Hooghly River (I1), within the patch of low surface EM conductivity (I4), and further east at shallow depths above the hot spot in As (I7) towards Chakdaha village (Fig. 5i). The first two of these profiles are also characterized by low major cation concentrations and elevated sulfate, but this not the case above the hot spot. Low sulfate concentrations and particularly elevated Fe(II)/Fe ratios at I5 show that low EM conductivity and low major cation concentrations are not always associated spatially with less reducing conditions in India.

The Old Brahmaputra appears to impact the nearby aquifer differently than the Hooghly River. Elevated Fe(II)/Fe ratios and low sulfate concentrations at B1 indicate particularly reducing conditions (Fig. 5h,j), even though major cation concentrations are relatively low. Further inland in Bangladesh, there is a mid-depth layer of mildly reducing conditions indicated by low Fe(II)/Fe ratios as well as a few samples elevated in sulfate at B2-B3.

**Figure 6:**
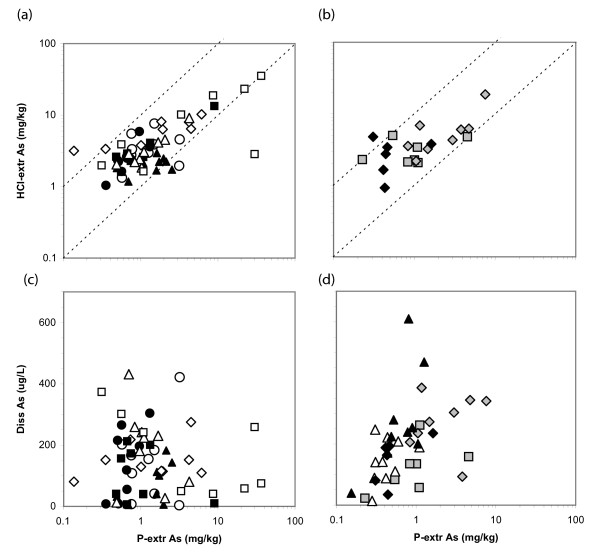
Comparison of P-extractable As concentrations with the acid-leachable As content of the sediment collected with from (a) Chakdaha, India, and (b) Balia, Para, Bangladesh. The two dashed lines in each panel correspond to a one-to-one correspondence and 10-fold higher HCl-extractable As concentrations, respectively. Also shown are dissolved As concentrations as a function of P-extractable As (c-d). Symbols identifying the profiles are the same as in Figure 2.

The range of HCl-extractable As concentrations in the sediment is comparable in India (1–35 mg kg^-1^) and Bangladesh (1–19 mg kg^-1^). But whereas HCl-extractable As concentrations in 6 out of 62 samples exceed 10 mg kg^-1 ^in India, this is the case for only 1 out of 22 samples in Bangladesh. There is only a very broad relationship between HCl and P-extractable As concentration in both India and Bangladesh (Fig. 6a,b). In the most enriched samples, collected from the eastern end of the transect in India (I7), concentrations of As in the P- and HCl-extractable fractions are comparable. At the low end of the spectrum, however, concentrations of As in the P-extractable fraction are up to an order of magnitude lower than in the fraction more aggressively leached with hot HCl. Because no HCl-extractable As data are available for 2 out of 5 profiles in Bangladesh, the discussion hereon focuses on the P-extractable As fraction.

The highest concentrations of P-extractable As are clustered towards the hot spot in India, with an average of 11 ± 14 mg kg^-1 ^(n = 9) over the entire depth range at I7 [See Additional file 2, Fig 4c]. The maximum in solid phase As is offset by one profile to the east relative to the highest dissolved As concentrations observed at I6, however (Fig. 5c). Concentrations of P-extractable As >5 mg kg^-1 ^are also observed in 2 samples below 15 m depth at both I4 and I5. In Bangladesh, elevated concentrations of P-extractable As are observed at several depth intervals at B3 only, i.e. approximately 80 m east of profile B2 where the highest dissolved As concentrations were measured [See Additional file 2, Fig 4]

## Discussion

### Redox conditions and groundwater recharge

In this section, geophysical and geochemical observations from India and Bangladesh other than As are first related to patterns of local vertical recharge recently documented within the broader area of Araihazar, which includes Balia Para. Consideration of lateral flow at depth towards discharge areas is beyond the scope of this study, especially because withdrawals by mechanized pumps in both India and Bangladesh are insufficiently documented.

Detailed surveys of surface lithology and EM conductivity, combined with profiles of groundwater age based on the ^3^H-^3^He dating technique, have shown that local vertical recharge is enhanced by an order of magnitude in areas where sandy deposits extend to the surface compared to areas where shallow aquifers are capped with less-permeable silts and clay [12-14]. Additional studies in the same area have shown that enhanced recharge where sandy deposits extend to the surface can prevent the shallowest aquifers from becoming very reducing, as indicated by Fe(II)/Fe ratios <0.5 [28,29]. The implication is that, at least in Araihazar, local recharge supplies an excess of electron acceptors over electron donors, presumably in the form of oxygen and nitrate. Enhanced vertical recharge therefore provides a plausible explanation for the association of low EM conductivity and relatively coarse-grained surface soils with low major cation concentrations and elevate sulfate levels in portions of the two new transects presented here (Fig. 3, Fig. 5g,h).

The geochemical characteristics of shallow aquifers closest to the Hooghly River appear to be controlled by local recharge. Low leachable Fe(II)/Fe ratios of 0.5 ± 0.06 to 30 m depth at I1 compared to the next profile further inland at I2 (0.68 ± 0.09), combined with Fe concentrations <0.1 mg L^-1 ^in most samples, suggests that recharge with river water prevents the sediment from becoming very reducing (Fig. 5e,i). Sulfate reduction appears to be limited at this location, as groundwater sulfate levels averaging 13 ± 3 mg L^-1 ^to 30 m depth are only slightly below the concentration of sulfate in river water in May 2005 (14 mg L^-1^). The presence of 0.3–2 mg L^-1 ^NO_3_^- ^in 4 out of 8 samples (Additional file 1) confirms a supply of electron acceptors favored over sulfate at I1. The groundwater collected at I1 was still anoxic at the time of sampling, however, as indicated by concentrations of Mn >0.1 mg L^-1^, at least 5 times higher than in filtered Hooghly River water (Additional file 1). It is striking that even though the signature of recharge extends to 30 m depth vertically at I1, elevated Fe(II)/Fe ratios and dissolved Fe concentrations combined with low sulfate indicate markedly more reducing condition at I2, which is located only 300 m east of I1. The satellite image of the area combined with the pattern of EM conductivities suggest that the thin layer of fine-grained material associated with the cultivated fields at I2 is sufficient to sharply restrict vertical recharge compared to I1, which is located on a coarser uncultivated deposit (Fig. 2a). The contrast maintained between I1 and I2 also suggests that local recharge of shallow aquifers is dominated by vertical rather than lateral flow, even along the banks of a major river such as the Hooghly.

Further inland in India, a relationship between recharge inferred from the surface lithology and redox conditions in underlying aquifers is more difficult to identify. Sandy deposits extend to the surface at I5 and not at I4, yet the second profile is actually closer to the area where the lowest EM conductivities were measured (Fig. 3). Cation concentrations are relatively low in shallow aquifers at both I4 and I5, but leachable Fe(II)/Fe ratios and sulfate concentrations indicate mildly reducing conditions in the subsurface at I4 (0.46 ± 0.07 and 14 ± 8 mg L^-1^, respectively) and more strongly reducing conditions at I5 (0.75 ± 0.13 and 3.17 ± 3.24 mg L^-1^). The expression of enhanced recharge associated with a supply of electron acceptors therefore appears to be restricted largely to I4, even though surface deposits are more permeable at I5. One possible explanation is that redox conditions at I5 are influenced by the presence of thicker impermeable deposits inferred from EM conductivity and located slightly to the north (Fig. 3c). Another factor could be proximity of I4 to the small stream that flows through the middle of the flooplain and could be a significant source of local recharge [21] (Fig. 2a). Whereas the various geochemical indicators consistently indicate a contrast in redox conditions between I4 and I5, unraveling the underlying hydrogeological factors at this small scale would require further study.

In Bangladesh, the Old Brahmaputra does not seem to have the same impact on the redox state of river bank aquifers as the Hooghly. The concentration of major cations is relatively low at B1, but elevated leachable Fe(II)/Fe ratios (0.8+0.14), low sulfate levels (0.5 ± 0.4 mg L^-1^), and elevated Fe concentrations (5.2 ± 1.2 mg L^-1^) all indicate strongly reducing conditions (Fig. 5f,h,j). Factors that may contribute to relatively reducing conditions near the Old Brahmaputra but cannot be distinguished on the basis of the available information include the low permeability surface soils at B1, the possibility that Old Brahmaputra River water turns anoxic during periods of stagnation in the dry season, and perhaps an excess of electron donors suggested by the color of the river. Further inland in Bangladesh, there are suggestions of an enhanced supply of electron acceptors even if sandy deposits extending to the surface were not observed at any of the drill sites. Cation concentrations are particularly low at B4 (3.3 ± 1.4 meq L^-1^) and sulfate levels are elevated in the shallowest intervals at the same location (Fig. 3h, Fig. 5h). Leachable Fe(II)/Fe ratios are also relatively low at B4, although the lowest values are observed mid-depth to the west at B2 and B3, along with occasionally elevated sulfate concentrations (Fig. 5g,i). These observations, along with the lower EM conductivity of inland soils, may indicate recharge from the large pond situated between B4 and B5, or by seasonal flooding of the entire low-lying, low EM conductivity area east of Balia Para.

### Redox conditions and partitioning of As

It is generally believed that As concentrations in shallow groundwater are regulated by adsorption onto reactive mineral surfaces rather than solubility with respect to a pure solid. Detailed spectroscopic investigations of aquifer material collected throughout the Bengal Basin indicate the presence of As in wide range of minerals, including micas, oxides, and sulfides, without any obvious association of a particular phase (or lack thereof) with elevated concentrations in groundwater [2,38]. Several studies have noted a significant correlation between the As and Fe content of aquifer particles, as well as an increase in concentrations of both with decreasing grain-size [2,3]. In a broad sense, it has also been shown that the As content of aquifer particles is lower in deeper strata that are typically associated with low As levels in groundwater [2,5,34,38]. To the best of our knowledge, however, sampling limitations have prevented a systematic comparison of groundwater As concentrations in shallow aquifers of the Bengal Basin with sediment properties from precisely the same horizon.

Even though the needle-sampler collects particles and groundwater from the same horizon, there still is remarkably little correspondence between the concentration of As in groundwater and the solid concentration of P-extractable As in India. Indeed, some of the highest As levels in the solid phase at I5 and I7 are associated with groundwater containing <100 μg L^-1 ^As and the three intervals containing >350 μg L^-1 ^As all contain sediment with <5 mg kg^-1 ^P-extractable As (Fig. 6c). The situation is different in Bangladesh, however, where increases in groundwater As concentrations are generally associated with higher P-extractable As levels [29]., with the exception of a few intervals at B3 and B5 (Fig. 6d). Unlike the situation in India, the relationship observed in Bangladesh suggests some form of exchange and equilibration of As between the solid and dissolved phase. Such a relationship was recently also documented on a regional scale in Bangladesh [39].

**Figure 7:**
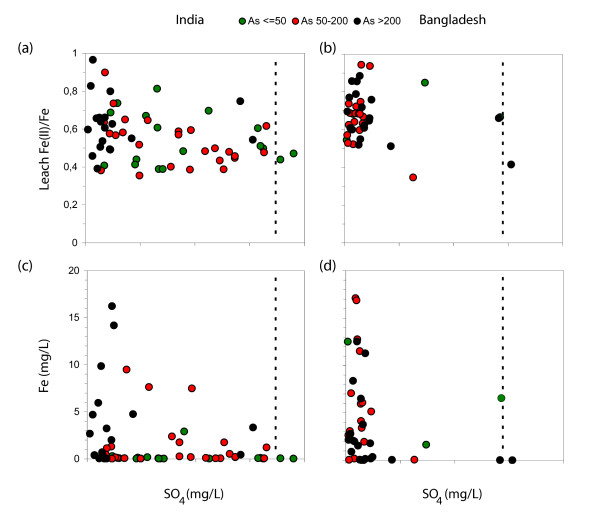
Comparison of sediment Fe(II)/Fe ratios (a-b) and dissolved Fe (c-d) concentrations as a function of sulphate for needle-sampler samples collected in India (left) and Bangladesh (right). The symbols are color-coded in three ranges according to groundwater As concentrations. Dashed lines show the sulfate concentration in the Hooghly and Old Brahmaputra rivers.

Why does there appear to be an association between P-extractable As and groundwater As in Bangladesh and not in India, given that the environments are broadly similar? The geochemical spectrum spanned by the composition of aquifer material in the two study areas differs in at least one major way. The high proportion of sediment samples in India with a leachable Fe(II)/Fe ratio <0.5 and elevated sulfate concentrations, combined with dissolved Fe concentrations <0.1 mg L^-1 ^at I1, I4, and I5 suggest the presence of Fe(III) adsorption sites for a sizeable subset of samples (Fig. 7a,c). More reducing conditions and low sulfate levels combined with generally elevated dissolved Fe concentrations in Bangladesh suggest instead a predominance of Fe(II) adsorption sites, probably in the form of oxyhydroxides [28,40] (e.g. magnetite) or possibly sulfides [41-43] (e.g. mackinawite) (Fig. 7b,d). We speculate that the proportion of groundwater samples containing <50 μg L^-1 ^As may be considerably higher in India than in Bangladesh despite higher P-extractable As levels because of a greater affinity of As for Fe(III) oxyhydroxides compared to Fe(II) oxyhydroxides or sulfides. The lack of strongly adsorbing Fe(III) oxyhydroxides in Bangladesh, and consequently perhaps more uniform adsorption properties for the remaining phases, could explain both why there are fewer samples containing <50 μg L^-1 ^As and why dissolved As concentration generally increase with the concentration of P-extractable As in the solid phase.

### Implications for the mechanisms As mobilization

Stute et al. [14] recently documented for the Araihazar area what may be the most systematic relationship reported to date between dissolved As concentrations and another property of shallow groundwater in the Bengal Basin. This is an increase of dissolved As concentrations with the age of groundwater determined by ^3^H-^3^He dating at a rate of approximately 20 ug/L As per year. Even though no age dating information is available, the general distribution of As in the shallow aquifers of Balia Para appears to be consistent with the As- age relationship in nearby villages. It is reasonable to expect that groundwater ages generally increase with depth along the B1-B5 transect, in parallel with the systematic increase of dissolved As. Reduced groundwater recharge over B2-B3, and therefore slightly higher ages compared to B4-B5, could plausibly be inferred from the pattern of EM conductivity, the location of a large pond, and the major cation content of shallow groundwater, and would be consistent with As concentrations at a given depth that generally decline east of B2 (Fig. 5d). At the western end of the transect on the banks of the Old Brahmaputra River (B1), an offset of the depth profile of As towards lower concentrations could also be attributed to lower groundwater ages compared to B2 on the basis of major cation concentrations.

Precipitation and river water contain little dissolved As and are likely to be associated with an excess of electron acceptors. Either dilution or stronger adsorption could therefore in principle maintain relatively low As concentrations in shallow aquifers containing relatively young groundwater. As previously noted in neighboring villages of Araihazar where groundwater ages were determined, however, there is no systematic relationship between As concentrations and redox conditions in Balia Para. Some of the highest As groundwater concentrations coincide with intervals showing elevated levels of sulfate and relatively low leachable Fe(II)/Fe ratios (Fig. 5d,h,j; Fig. 7b). The new data presented here confirm that reducing conditions seem to be a pre-condition for As release to groundwater but are not sufficient to explain all the patterns observed along the transect in Bangladesh.

Even though no ^3^H-^3^He data are available for Chakdaha, or to the best of our knowledge for any site in the western portion of the Bengal Basin, the existing information suggests that a consistent relationship between groundwater ages and dissolved As concentrations is unlikely. The profile at I1 is a case in point. Location near a major river, a relatively permeable surface soils, low major cation concentrations, and elevated sulfate levels all suggest the presence of groundwater that was recently recharged (Fig. 3c,g). And yet, some of the highest dissolved As concentrations were observed in the shallowest aquifers at this very location (Fig. 4a; Fig. 5c). Further inland at I4 as well, the combination low EM conductivity, low major cation concentrations, and elevated sulfate concentrations would suggest relatively young groundwater even though As concentrations exceed 150 ug/L in three intervals between 9 and 15 m depth.

Why is the distribution of As beneath the floodplain of the Hooghly particularly variable and seemingly unrelated to processes that have been shown to play an important role in Bangladesh? One possible explanation is that the supply of large loads of sediment to the study area in Araihazar was cut off a few centuries ago [12]. Over this period, groundwater flow could have dampened some of the heterogeneities in the initial distribution of As. Release, transport, and re-adsorption could have redistributed As within the aquifers in away that reflects some form of partitioning between the dissolved and particulate phase [39]. Finally, flushing of As along preferential flow paths could over time could have produced the observed relationship between As concentrations and groundwater age [14]. In contrast to the Old Brahmaputra River, accumulation of sediment is likely to be still very significant along the floodplain of a major stream such as the Hooghly River. Although the shallow deposits west of Chakdaha have not been dated, they probably are considerably younger than in Araihazar [12,34]. Differences in the carbonate chemistry of groundwater provide circumstantial evidence of a difference in maturity of the two study sites (Additional file 1). With the exception of I1 which is indistinguishable from Hooghly River water (205 ± 11 μmol/L, n = 9 vs. 228 μmol/L, respectively), the bicarbonate content of groundwater collected with the needle-sampler was almost always considerably higher in India (503 ± 91 μmol/L, n = 54) compared to Bangladesh (295+37 μmol/L, n = 22). Whereas several other scenarios are possible, this could be interpreted as an indication that more reactive organic matter is metabolized in Hooghly floodplain deposits because of its younger age. If confirmed, the on-going supply of freshly-eroded sediment could explain a more heterogeneous distribution of As, the lack of a systematic relationship between As concentrations in the dissolved phase and solid phase, and may result in the release of As through additional processes. At I1 near the Hooghly River and further inland at I4, for instance, As could plausibly be released by oxidation of pyrite grains transported from the Himalayas rather than reduction of Fe oxyhydroxides. This mechanism has been discounted as the leading cause of groundwater As enrichments throughout the Bengal Basin [2,5], but as recently proposed, could still play an important role in young deposits at shallow depth [11]. An alternative explanation for the differences between Chakdaha and Balia is that sediment contributed by the Hooghly and Old Brahmaputra River (actually its larger precursor) are sufficiently different nature in terms of As concentrations or As and/or Fe mineralogy to generate different patterns of release. We have no data to evaluate this possibility, but note that attempts to link groundwater As concentrations within Holocene aquifers to mineralogy have not been successful [2].

### Implications for future studies

Previous field and laboratory investigations have given different weight to the relative importance of various geological, hydrological, geochemical, microbial factors for the release of As to groundwater of the Bengal Basin. This is the natural reflection of the different spectrum of skills mastered by individual teams and various geographical constraints. Probably by circumstance rather than by design, some areas of investigation such as organic geochemistry [44] and sulfur geochemistry [45,46] have until recently been often overlooked. To properly address the complexities made apparent by the present study and previous work, and by analogy to large projects in high-energy physics, a highly interdisciplinary team of scientists may have to be assembled to jointly study a limited number of carefully selected sites for an extended period of time. Because of the importance of recharge and flow patterns, it would be essential to involve some of the handful of laboratories with the capacity to measure ^3^H-^3^He ages. The contrasting conditions encountered at Chakdaha and Araihazar indicate that constraints on sediment age using radiocarbon, ^210^Pb, and optically-stimulated luminescence should also be an integral part of such an effort [12,34].

## Conclusion

On the basis of an unusually dense array of matched samples of groundwater and aquifer solids from two different study areas, the present study shows that the distribution of As in shallow groundwater of the Bengal Basin is likely to be controlled by multiple processes. Although recharge was not measured, it appears to play a particularly important role through dilution with As-depleted water and an associated input of electron acceptors that favors adsorption of As. The new data from widely separated portions of the Bengal Basin are consistent with Fe oxyhydroxide reduction as a source of As, but also suggest other potential mechanism that may be important in relatively young deposits. The limitations of the present study suggest that the detailed views of the subsurface afforded by the needle-sampler need to be systematically supplemented with data constraining the age of sediment as well as groundwater.

## Competing interests

The authors declare that they have no competing interests.

## Authors' contributions

JM, MWR, and SBM carried out the field-work in India. JM, MWR, and KMA carried out the field work in Bangladesh. JM, SB, and ZC measured the composition of groundwater and sediment leachates in the laboratory. LC and AvG conceived of the study and helped JM write the manuscript. All authors read and approved the final manuscript.

## Supplementary Material

Additional file 1 Geochemical and geophysical data from India and Bangladesh. The worksheets of this spreadsheet include a listing of (1) latitude and longitude of all needle-sampler profiles, (2) needle-sampler data for India, (3) well data for India, (4) EM31 data for India, (5) needle-sampler data for Bangladesh, and (6) EM31 data for Bangladesh. Click here for file

Additional file 2 Supplemental figures with legends. Click here for file
