# Supplementary material for: Comparison of dissolved and particulate arsenic distributions in shallow aquifers of Chakdaha, India, and Araihazar, Bangladesh
Source: Geochem Trans. 2008 Jan 11;9:1. doi: 10.1186/1467-4866-9-1 (PMC2246114; doi:10.1186/1467-4866-9-1)
Supplement: Additional file 2 — Supplemental figures with legends. [file 1467-4866-9-1-S2.pdf]

Figure S1

Comparison of sulphate concentrations measured by HR ICP-MS and ion chromatography.

Figure S2

Comparison of composition of 21 “replicate” needle-samples collected sequentially 1-3 ft apart in India and Bangladesh. Symbols identifying the profiles are the same as in Figure 2.

Figure S3

Comparison of conductivity and the major ion content of groundwater samples collected with the needle-sampler.

Figure S4

Contoured sections of As concentrations in the HCl-leachable (a-b) and P-extractable fraction (c-d) in the solid phase based on the needle-sampler profiles.

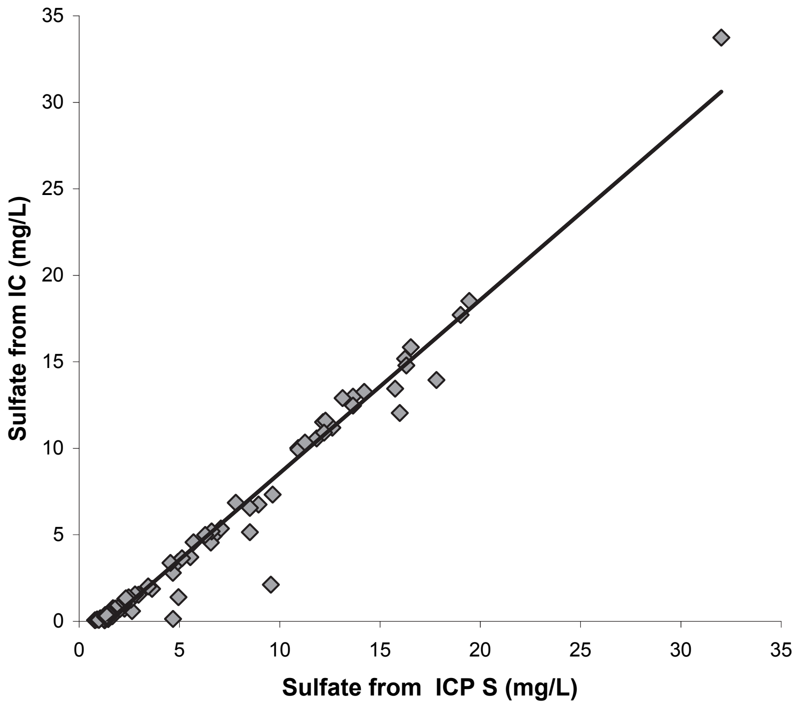

Figure S1

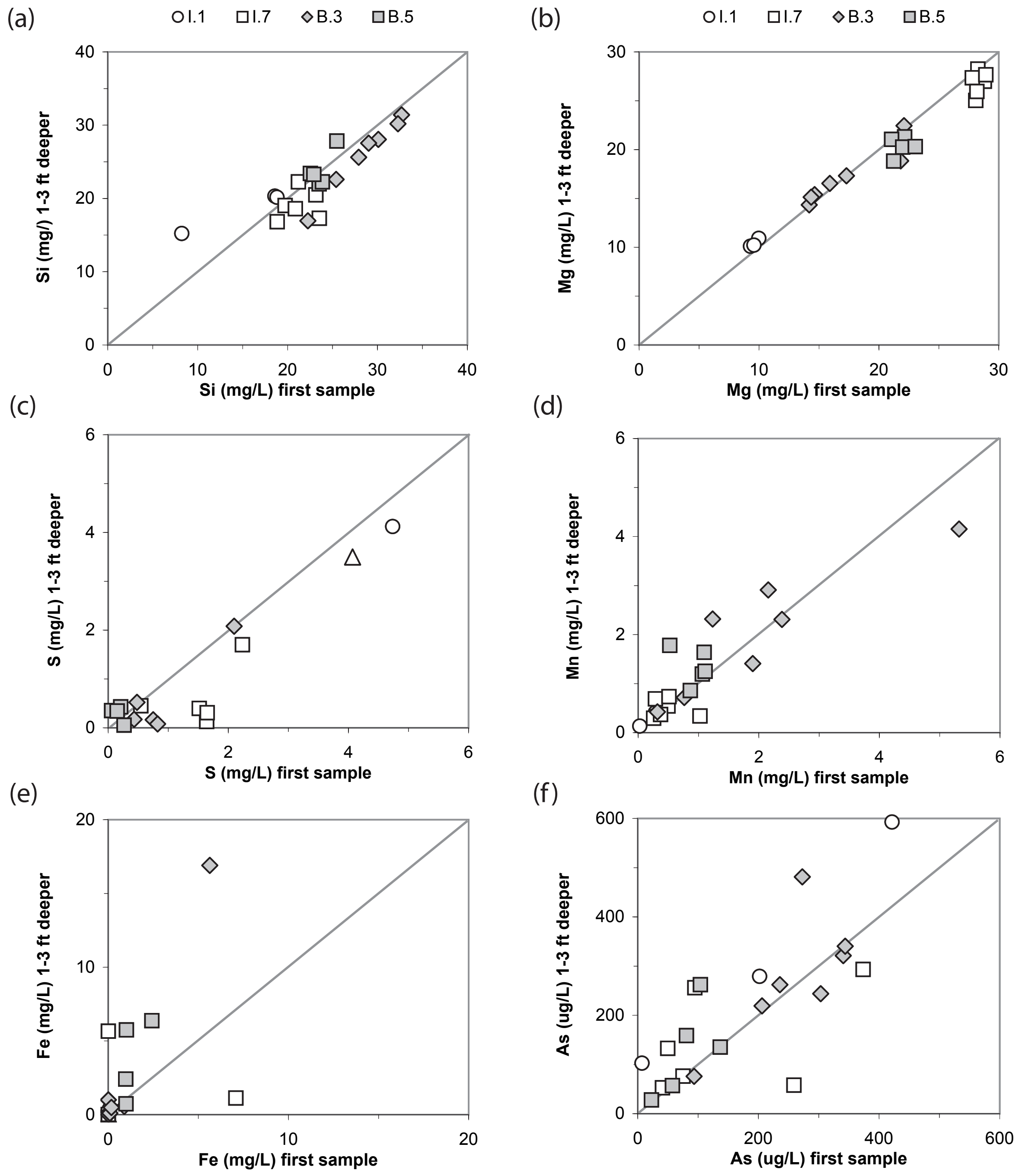

(a)
○I.1 □I.7 ◇B.3 ■B.5
Si (mg/) 1-3 ft deeper
Si (mg/L) first sample
(b)
○I.1 □I.7 ◇B.3 ■B.5
Mg (mg/L) 1-3 ft deeper
Mg (mg/L) first sample
(c)
S (mg/L) 1-3 ft deeper
S (mg/L) first sample
(d)
Mn (mg/L) 1-3 ft deeper
Mn (mg/L) first sample
(e)
Fe (mg/L) 1-3 ft deeper
Fe (mg/L) first sample
(f)
As (ug/L) 1-3 ft deeper
As (ug/L) first sample

Figure S2

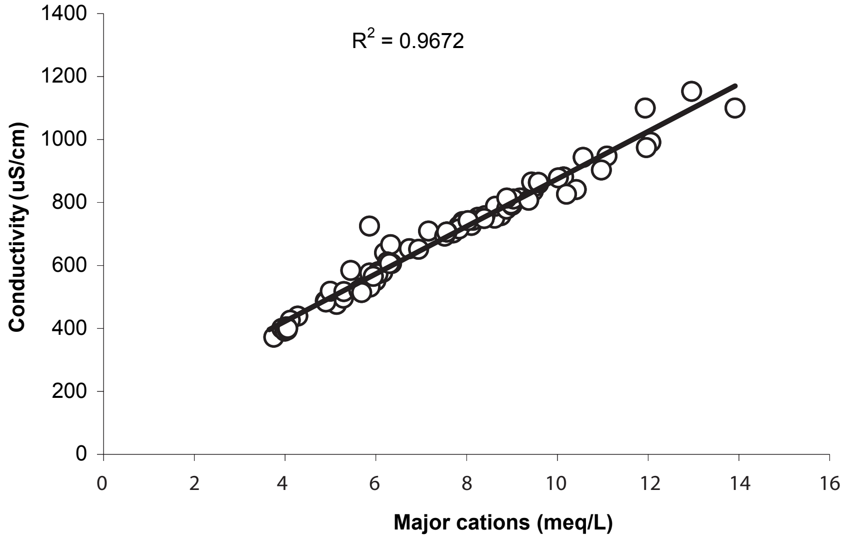
R2 = 0.9672
Conductivity (uS/cm)
Major cations (meq/L)
1400
1200
1000
800
600
400
200
0
0
2
4
6
8
10
12
14
16

Figure S3

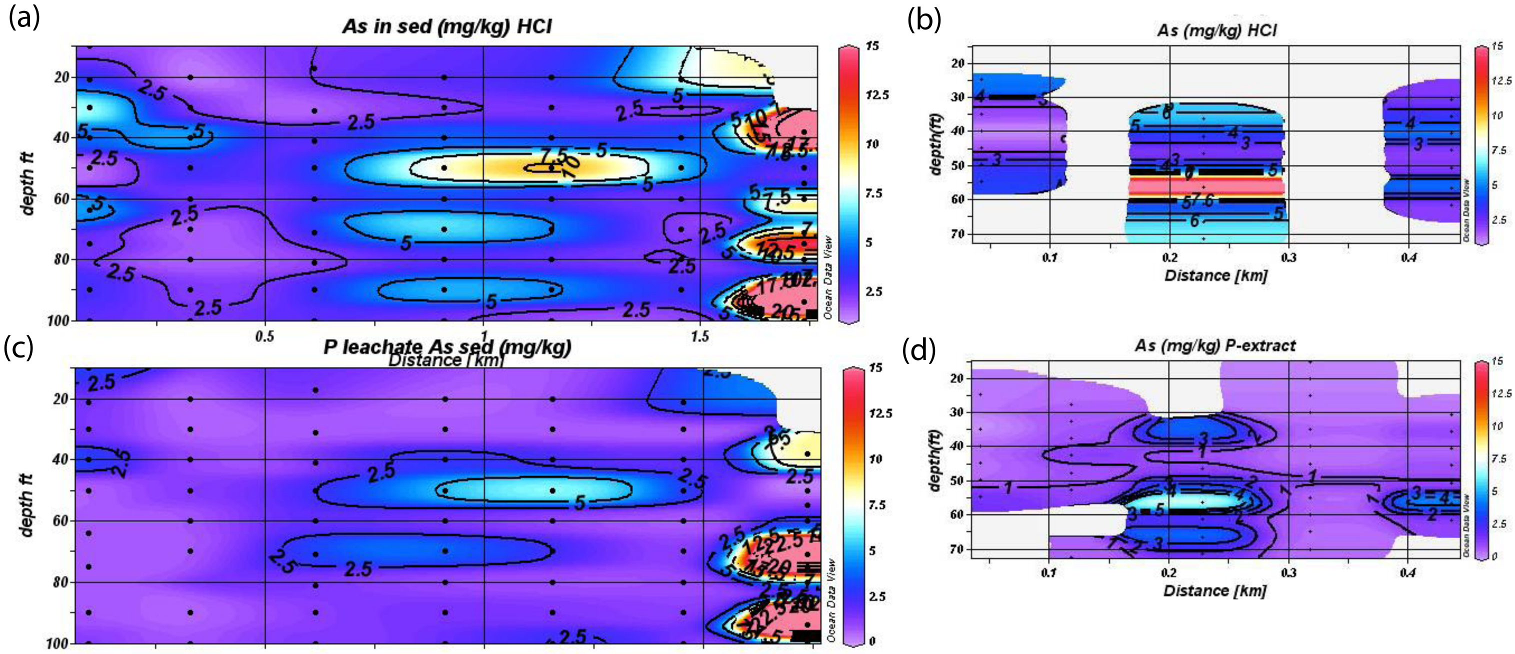
(a)
As in sed (mg/kg) HCl
depth ft
Distance [km]
Ocean Data View
(b)
As (mg/kg) HCl
depth(ft)
Distance [km]
Ocean Data View
(c)
P leachate As sed (mg/kg)
depth ft
Ocean Data View
(d)
As (mg/kg) P-extract
depth(ft)
Distance [km]
Ocean Data View

Figure S4
